# Schizophrenia and Leigh syndrome, a simple comorbidity or the same etiopathogeny: about a case

**DOI:** 10.11604/pamj.2015.22.333.8288

**Published:** 2015-12-04

**Authors:** Leila Mnif, Rim Sellami, Jawaher Masmoudi

**Affiliations:** 1 Department of Psychiatry «A», Hédi Chaker University Hospital, Sfax, Tunisia

**Keywords:** Leigh syndrome, psychiatric disorders, schizophrenia

## Abstract

Leigh syndrome is a mitochondrial encephalomyopathy that occurs due to “cytochrome c oxidase deficiency”. Few psychiatric disorders have been defined that are associated with Leigh syndrome. The objective of this work is to study relations between mitochondrial dysfunction and psychiatric disorders. It was a 20 year old male patient, who received Modopar, for severe extra pyramidal symptoms caused by Leigh syndrome. He developed, four months ago, acute psychotic symptoms such as audio-visual hallucinations, persecution and mystic delirium. The cerebral MRI has shown signal abnormalities in central grey nucleus. The EEG recording and blood test were normal. The hypothesis of drug induced psychiatric disorders (Modopar) was possible. The evolution under atypical antipsychotic was only partial. In this case, the cerebrospinal fluid and lactate levels mean that mitochondria were not an overall explanation for these psychiatric disorders but may at least play a partial role. Psychiatric disorders may just be acomorbidity.

## Introduction

Leigh syndrome is a progressive neurodegenerative disorder with complication by episodes of lactic acidosis of childhood with an estimated incidence of 1:40,000 births [1]. Clinically, Leigh syndrome is characterized by psychomotor delay or regression, muscular hypotonia, brainstem signs (especially strabismus, nystagmus and swallowing difficulties), ataxia, pyramidal signs, respiratory insufficiency, lactate acidemia and acute deterioration after common infections. In most cases, dysfunction of the respiratory chain enzymes is responsible for the disease, which could be due to defects in either mitochondrial or nuclear DNA. Few reports [2, 3] mention psychiatric disorders. The comorbidity in the psychotic symptoms seen in both mitochondrial and psychiatric diseases is what triggered an interest in the effects of mutations of mtDNA and psychological disorders [4, 5]. So, evidence is accumulating that mitochondrial dysfunction is involved in the pathophysiology of some psychiatric disorders such as Bipolar Disorder and Schizophrenia. In addition, among other symptoms, people diagnosed with mitochondrial disease have high rates of psychiatric disorders. The objective of this work is to study relations between mitochondrial dysfunction and psychiatric disorders through a case of Leigh Syndrome.

## Patient and observation

A 20 year-old man, child of second degree consanguineous marriage presented to our consultation for behavioral disorders. At age 5, our patient lost slowly the ability to walk. He has been taking L-Dopa, for severe extra pyramidal symptoms caused by Leigh syndrome. It has been the source of stopping his studies at the secondary level, due to the difficulties in speech and movement. The diagnosis of Leigh syndrome was based on clinical, radiological and biographical indices, such as: axial and peripheral extra pyramidal syndrome, dystonia of the members, rotatory nystagmus, signal abnormalities in the central gray nucleus at the cerebral magnetic resonance imaging (MRI) and the notion of death of two sisters in infancy; because of evolutionary consequences of Leigh syndrome. He has no history of addiction or alcohol consumption. The assessment of his cognitive function was normal. Genetic study cannot be realized because the unavailability of appropriate techniques in Tunisia. He was described as an, emotionally cold person, and known for an increased interest for studies relative to leisure.

Two years ago, suddenly and without triggering factor, the patient developed soliloquy, verbal aggressiveness to his parents, persecution, mystical talk and sleep disturbance. The physical examination found a patient moving in a wheelchair, having regard to functional impairment secondary to the severity of extrapyramidal syndrome in his limbs. His conscience was clear, without any confusion. There was rotatory nystagmus and dystonia of the hands and feet. The psychiatric examination revealed, a young white man unable to maintain good contact, but who appeared anxious and suspicious. His speech is poor in content, barely understandable because of dysarthria. He reports a fuzzy; non systematized delusion: He was convinced that he was the prophet “Noah ” and was charged with a divine mission: to change the world functioning. He is persecuted by strangers who collaborate with his parents to kill him. He experienced auditory and visual hallucinations. Specialized neurological examination did not showed any clinical worsening. The physical examination was normal. Investigations included routine blood tests (CBC, blood sedimentation reaction (BSR), liver and renal function assessments, serum electrolytes, an electroencephalogram, which were normal. MRI showed signal abnormalities in the central gray nucleus (basal ganglia) which was similar to those observed in the MRI practiced five years ago. The cerebrospinal fluid (CSF) and blood lactate levels were normal.

We prescribed amisulpiride in small doses (300mg/day) combined with a benzodiazepine (Lorazépam: 7,5 mg/day) and a corrector of extrapyramidal effects (biperiden hydrochloride). The use of higher doses of antipsychotic had led to as significant exacerbation of side effects. The partial remission could be observed under these doses, but quickly followed by a relapse during treatment, after 4 months, with exacerbation of positive symptoms and appearance of bizarre behavior. This relapse had necessitated a switch to aripiprazole at a dose of 30 mg / day achieved gradually. This drug was well tolerated, and any neurological deterioration was found. This fact allowed us to decrease and stop antiparkinsonian drugs (L-Dopa and biperiden hydrochloride). But we opted, after 6 weeks of treatment, for the addition of valproic acid (1500 mg/day) and clonazepam (4mg/day) such as a strategy of potentiating, given the persistence of positive symptoms, the anxiety and behavioral corollaries, such as distrust of his parents, the refusal of any food prepared by them, and insomnia. Clinical improvement was very partial to afford acceptable interfamily adaptation, after 6 months of treatment. A third switch to olanzapine at a dose of 15 mg / day was made, while maintaining of valproic acid and clonazepam. This combination allowed a partial remission, allowing a best level of adaptation, it has been maintained. The clozapine has not been tried in this case.

## Discussion

This case illustrates the presentation of Schizophrenia characterized by the acute onset of delusional and hallucinatory syndrome. There are elements of negative prognosis for schizophrenic evolution: the absence of triggering factor, a poverty of delusion, schizoid personality and the partial improvement with antipsychotic.

This diagnosis can only be made after other causes have been excluded specifically toxic cause, metabolic origin (lactic acidosis secondary to mitochondrial dysfunction): but blood gas, CSF and blood lactate levels are normal. A brain tumor was eliminated by brain MRI. The normality of the EEG and the seat of brain lesions on MRI made the diagnosis of partial epilepsy unlikely. However, psychiatric disorders induced by the drug (L-Dopa) were possible. But, the persistence of symptoms despite the dropping of medicine is not in favor of the diagnostic. The normality of explorations raises the question of relationship between the two diseases: Psychiatric disorders may be just a simple comorbidity or are secondary to the Leigh Syndrome? William Regenold and coll [6] said people diagnosed with mitochondrial disease have high rates of psychiatric disorders or symptoms such as depression, visual hallucinations, bipolar disorder or anxiety. They speculated that some psychiatric diseases might be related to glucose processing problems involving mitochondria. They measured levels of lactate; a product of extra mitochondrial glucose metabolism; in two groups of 15 patients, each with schizophrenia or bipolar disorder and also healthy controls. Significantly high concentrations were found in bipolar and schizophrenic groups, compared with healthy controls. For them, the CSF lactate levels are not an overall explanation for those psychiatric disorders but may at least play a partial role. Furthermore, Regenold et al. [6] considered possible effects of medication or heredity. A number of psychotropic drugs, such as haloperidol and clozapine, shows anti mitochondrial effects.

In their study, the researchers found only a trend toward lower CSF lactate concentration (p = 0.058) associated with any antipsychotic use. Although many questions about the connection between CSF lactate concentrations and psychiatric disorders remain. On autopsy, brain tissue of schizophrenic patients shows a reduction in the number and density of mitochondria in the frontal cortex, striatum, and substantia nigra. Interestingly, when divided into drug-off and drug-on groups, the patients who were on antipsychotic treatment had more normal mitochondrial density and morphology [7]. There is also strong evidence, in vivo and in vitro, that dopamine, long known to initiate psychotic symptoms, plays a predominant role in the mitochondrial respiratory system of patients suffering from schizophrenia [8]. However, studies have come to the conclusion that mitochondrial dysfunction maybe the primary origin in this disorder. On the other hand, Halim and coll [9] studied the pH and the lactate levels in post-mortem brains of schizophrenics. They suggest that lactate increases are possibly related to antipsychotic treatment rather than to a primary metabolic abnormality. Alterations in mitochondrial function were found in the prefrontal cortex of post mortem schizophrenics. The prefrontal cortex is especially important with schizophrenia because it is part of both the mesolimbic and mesocortical pathways in the brain. These pathways have implications in the emergence of positive and negative symptoms respectively of the disorder. Although mitochondrial diseases manifest themselves in largely physical symptoms, it has been reported that individuals with these disorders display a high prevalence of psychiatric symptoms including auditory and visual hallucinations, delusions, erratic behavior, and depression. Some patients have been diagnosed with psychosis and schizophrenia; others have found that the diagnosis of the psychiatric disease actually obscured the additional diagnosis of mitochondrial disorders for up to a decade.

Significant decrease in mitochondria number and density in the prefrontal cortex and caudate nucleus of postmortem brains of subjects with Schizophrenia was observed compared with control subjects [10]. A lower number of mitochondria in patients who were not taking antipsychotic medications were found compared with those taking antipsychotics or control medications, suggesting drug treatment normalized the number of mitochondria [11]. In fact, neuroleptic medications have been reported to have inhibitory effects on mitochondrial function [12, 13]. The fact that atypical antipsychotics including quetiapine have negligible inhibitory effects of mitochondrial oxidative phosphorylation provides a potential benefit on mitochondrial respiration when compared to typical antipsychotics. The cost of this drug was very expensive, for the family of the patient.

Indeed, Therapy of psychosis in patients with mitochondrial disorders is principally not at variance from treatment of psychosis in non-mitochondrial patients [14]. There is no consensus on the therapy of psychosis in patients with mitochondrial disorder [14], whereas it was resistant forms of schizophrenia.

## Conclusion

In this case, the CSF lactate levels mean that mitochondria is not an overall explanation for these psychiatric disorders but may at least play a partial role, raising the question of relationship between the two diseases: Psychiatric disorders may be just a simple comorbidity or are secondary to the Leigh Syndrome. It is possible that the new data will lead to a focus on psychiatric disorder as a metabolic. The future study of mitochondrial dysfunction in psychiatric disorders should bring clarification as to how nonspecific mitochondrial dysfunction can cause specific symptoms of psychiatric disorders. Further study of mitochondrial dysfunction in psychiatric disorder is expected to be useful for the development of cellular disease markers and new psychotropic.
